# Therapeutic Application of Micellar Solubilized Xanthohumol in a Western-Type Diet-Induced Mouse Model of Obesity, Diabetes and Non-Alcoholic Fatty Liver Disease

**DOI:** 10.3390/cells8040359

**Published:** 2019-04-17

**Authors:** Abdo Mahli, Tatjana Seitz, Kim Freese, Jan Frank, Ralf Weiskirchen, Mona Abdel-Tawab, Dariush Behnam, Claus Hellerbrand

**Affiliations:** 1 Institute of Biochemistry (Emil-Fischer Zentrum), Friedrich-Alexander University Erlangen-Nürnberg, Fahrstr. 17, D-91054 Erlangen, Germany; Abdo.Mahli@fau.de (A.M.); Tatjana.Seitz@fau.de (T.S.); Kim.Freese@fau.de (K.F.); 2 Institute of Nutritional Sciences, University of Hohenheim, Garbenstr. 28, D-70599 Stuttgart, Germany; jan.frank@nutres.de; 3 Institute of Molecular Pathobiochemistry, Experimental Gene Therapy and Clinical Chemistry (IFMPEGKC), RWTH University Hospital Aachen, D-52074 Aachen, Germany; rweiskirchen@ukaachen.de; 4 Central Laboratory of German Pharmacists, Carl-Mannich-Str. 20, D-65760 Eschborn, Germany; m.tawab@zentrallabor.com; 5 AQUANOVA AG, Birkenweg 8-10, D-64295 Darmstadt, Germany; Dariush.Behnam@aquanova.de

**Keywords:** xanthohumol, micellar solubilisation, obesity, diabetes, non-alcoholic fatty liver disease

## Abstract

Xanthohumol (XN), a prenylated chalcone from hops, has been reported to exhibit a variety of health-beneficial effects. However, poor bioavailability may limit its application in the prevention and therapy of diseases. The objective of this study was to determine whether a micellar solubilization of xanthohumol could enhance the bioavailability and biological efficacy of xanthohumol in a Western-type diet (WTD) induced model of obesity, diabetes and non-alcoholic fatty liver disease (NAFLD). After 3 weeks feeding with WTD, XN was additionally applied per oral gavage as micellar solubilizate (s-XN) or native extract (n-XN) at a daily dose of 2.5 mg/kg body weight for a further 8 weeks. Control mice received vehicle only in addition to the WTD. WTD-induced body weight-gain and glucose intolerance were significantly inhibited by s-XN application. Furthermore, WTD-induced hepatic steatosis, pro-inflammatory gene expression (MCP-1 and CXCL1) and immune cell infiltration as well as activation of hepatic stellate cells (HSC) and expression of collagen alpha I were significantly reduced in the livers of s-XN-treated mice compared to WTD controls. In contrast, application of n-XN had no or only slight effects on the WTD-induced pathological effects. In line with this, plasma XN concentration ranged between 100–330 nmol/L in the s-XN group while XN was not detectable in the serum samples of n-XN-treated mice. In conclusion, micellar solubilization enhanced the bioavailability and beneficial effects of xanthohumol on different components of the metabolic syndrome including all pathological steps of NAFLD. Notably, this was achieved in a dose more than 10-fold lower than effective beneficial doses of native xanthohumol reported in previous in vivo studies.

## 1. Introduction

The term “metabolic syndrome” refers to the simultaneous occurrence of several comorbidities associated with obesity. In addition to insulin resistance with resulting type 2 diabetes mellitus (T2DM), dyslipidemia and hypertension are components of the metabolic syndrome. Moreover, in almost all cases, obesity is associated with significant lipid accumulation in the liver. This form of hepatic steatosis is defined as nonalcoholic fatty liver disease (NAFLD) and is today regarded as the hepatic manifestation of the metabolic syndrome. NAFLD encompasses a wide range of pathological conditions. Hepatocellular lipid accumulation is the first pathological step of NAFLD. Simple steatosis can progress with inflammation to (non-alcoholic) steatohepatitis (NASH). In its advanced form, NASH is characterized by necroinflammation and progressive fibrosis [1]. The latter can progress to cirrhosis. Today, NAFLD is recognized as the most common liver disease in Western countries [2].

Each pathological NAFLD step is a consequence and triggered by (components of) the metabolic syndrome, respectively. Herewith, improving (components of) the metabolic syndrome also improves NAFLD.

Currently, there are no efficient strategies available for the treatment of NAFLD. The majority of patients do not respond well to weight-loss interventions [3]. Surgical interventions such as gastric bypass or gastric downsizing (bariatric surgery) are the most effective measures for permanent weight loss [4], which also positively influence NAFLD development and progression [5]. However, also in light of the invasiveness and costs, these surgical interventions are only applicable for a minority of pathologically obese individuals. On the other hand, pharmacological therapies for T2DM (for example with sulfonylurea, metformin, or GLP-1 agonists) are often only partially effective and suffer from severe side effects [6]. Therefore, there is a high clinical need for new pharmacological therapeutic approaches for sustainable safe weight loss and for long-term improvement of (symptoms of the) metabolic syndrome.

Xanthohumol (3’-[3,3-dimethyl allyl]-2’,4’,4-trihydroxy-6’-methoxychalcone) is the main prenylated chalcone of the female inflorescences (hop cones), present in small amounts in beer [7]. Several health-beneficial effects of xanthohumol have been reported such as chemopreventive and anti-inflammatory activities (reviewed in [8]). More recently, xanthohumol has been shown to decrease adipogenesis and to improve lipid and glucose metabolism in murine models of hyperlipidemia, obesity and T2DM [9,10,11]. Moreover, we have shown previously that xanthohumol inhibits hepatic inflammation and fibrosis in murine models of NAFLD [12]. Furthermore, we and others found that xanthohumol inhibits hepatic inflammation and fibrosis also in models of hepatocellular injury not related to the metabolic syndrome [13,14,15]. This indicates that xanthohumol also exhibits direct inhibitory effects on inflammatory and fibrogenic mechanisms in the liver (cells).

It is noteworthy that in all these studies, XN was administered from the beginning of the experiment, i.e., simultaneously with the introduction of an obesogenic or NAFLD-inducing diet, which simulates disease prevention. This, however, does not reflect the situation of obese patients, who already suffer from medical conditions upon commencement of ameliorating interventions. Furthermore, and despite its promising health effects, the therapeutic use of XN may be limited by its poor oral bioavailability [16].

In the present study, we aimed to analyze the effect of a micellar solubilization of xanthohumol (s-XN) in a preclinical mouse model of diet-induced obesity, diabetes and NAFLD [17]. Based on previous studies successfully using micellar solubilization of other polyphenols to enhance their oral bioavailability [18,19,20,21], we chose a daily dose as low as 2.5 mg/kg BW, i.e., more than 10-fold lower than effective doses reported in previous in vivo studies. For comparison, we also applied the same dose of xanthohumol in its native form (n-XN). Different than in most previous studies, xanthohumol was not administered via (mixing into) chow but via daily oral gavage to guarantee exact dosing. Furthermore, mice were fed with an obesity- and NAFLD-inducing Western-type diet (WTD) already 3 weeks before the beginning of xanthohumol administration to simulate therapeutic intervention rather than prevention.

## 2. Materials and Methods

### 2.1. Chemicals

Micellar Xantho-Flav-Solubilisate (EW0192/B, 10% *v*/*v*; s-XN) and native xanthohumol extract (92% *m*/*m*; n-XN) were provided by AQUANOVA AG (Darmstadt, Germany). For dissolution of native xanthohumol, an aqueous solution of methyl hydroxypropyl cellulose (MHPC, 0.2%, Methocel E4M Premium CR, Hypromellose 2910 USP, Fagron Inc., St. Paul, MN, USA) was prepared.

### 2.2. Animals and Animal Model

Eight-week-old male C57BL/6 mice (Charles River Laboratories; Sulzfeld, Germany). Mice were housed in a 22 °C controlled room under a 12-h light–dark cycle with free access to food and water. Housing and animal experiments were performed according to national and international guidelines of the European Union (EU) with the appropriate permission of the local government (55.2-2532-2-196). After 1 week of acclimatization, mice were divided into four groups (6 mice per group); mice were fed either a standard diet (control) or a Western-type diet (WTD) for three weeks. Standard diet contained 4.9% crude fiber, 6.4% crude ash, 19% crude protein, 3.3% fat and 58% carbohydrates (sucrose 4.7% and starch 36.5%); WTD was enriched with pork lard (15%), beef tallow (15%), palmitic acid (4%), stearic acid (4%), cholesterol (0.2%) and sucrose (30%) [17]. Both rodent diets were prepared by Ssniff (Soest, Germany).

Subsequently, WTD-fed mice were treated with either n-XN or s-XN or vehicle (VH) per oral gavage daily for an additional 8 weeks. After a total of 11 weeks of control or WTD-feeding, mice were sacrificed and liver tissue and blood samples were collected for further analysis.

### 2.3. Intraperitoneal Glucose Tolerance Test (ipGTT)

After 7 weeks of oral administration of n-XN or s-XN, ipGTT was performed as described previously [22]. Briefly, after 12-h fasting, mice were injected intraperitoneally with glucose (50% *w*/*v* solution, 3 mg (glucose)/g body weight). Blood glucose concentrations were measured in samples taken from the tail vein before (fasting glucose) and 30, 60, 90 and 120 min after glucose administration using an Accutrend glucometer (Roche, Mannheim, Germany).

### 2.4. Analysis of Cellular Lipid Content

Hepatic lipids were extracted and triglyceride levels were quantified using the GPO-triglyceride kit (Sigma, Deisenhofen, Germany) as described [23,24].

### 2.5. RNA Expression Analysis

RNA isolation from liver tissues and reverse transcription were performed as described [25]. Quantitative real-time-PCR was performed applying LightCycler technology (Roche) [26] and the following pairs of primers: Murine collagen I (forward: 5′-CTG TTC CAG GCA ATC CAC GA -3′; reverse: 5′-ATC AGC TGG AGT TTC CGT GC-3′), murine cxcl1 (forward: 5′-ACC GAA GTC ATA GCC ACA CTC-3′; reverse:5′-CTC CGT TAC TTG GGG ACA CC-3′), murine mcp1 (forward: 5′-TGC AGG TCC CTG TCA TGC TTC-3′; reverse: 5′-TGG ACC CAT TCC TTC TTG GGG T-3′), and murine α-SMA (forward: 5′-CCA GCC ATC TTT CAT TGG GAT-3′; reverse: 5′-CCC CTG ACA GGA CGT TGT TA-3′). Amplification of cDNA derived from 18s rRNA (forward: 5′-AAA CGG CTA CCA CAT CCA AG-3′; reverse: 5′-CCT CCA ATG GAT CCT CGT TA-3′) was used for normalization.

### 2.6. (Immuno) Histological Analyses

For histological analyses, murine liver tissue specimens were fixed for 24 h in 4% formalin at room temperature, dehydrated by graded ethanol and embedded in paraffin. Tissue sections (thickness 5 µm) were deparaffinized with xylene and stained with haematoxylin and eosin. Immunohistochemical staining was performed using anti-CD3 C7930 antibody (1:500; Sigma-Aldrich) as described [27]. The number of CD3-positive hepatocytes was counted in 4 randomly selected areas on each section. Hepatic immunohistochemical staining for α-SMA was performed as described [17].

### 2.7. HPLC Analysis of XN in Serum Samples

Xanthohumol was analyzed as previously described [7] with small modifications to allow the use of larger volumes of serum due to low blood concentrations of the analytes. Briefly, serum samples, in duplicate, were adjusted to pH 4.5–5.0 with 6 N hydrochloric acid. Ascorbic acid (12.5 μL of 10% ascorbic acid in water; wt/vol) was added to prevent oxidation. Serum (1.5 mL) was incubated with 25 μL β-glucuronidase type H-1 from Helix pomatia (3 mg/100 µL in 0.1 M sodium acetate buffer, pH 4.5; Sigma-Aldrich Chemie GmbH, Schnelldorf, Germany) in the dark at 37 °C for 1 h with gentle shaking (~100 rpm). Samples were extracted twice with 4 mL ethyl acetate by mixing for 20 min on a vertical rotator. Samples were centrifuged at 1690× *g* for 5 min at 4 °C in between mixing and a total of 7.2 mL of supernatant was recovered. The pooled supernatants were evaporated to dryness at 10 mbar using an RVC 2-33 IR centrifugal evaporator (Martin Christ Gefriertrockungsanlagen GmbH, Osterode am Harz, Germany). Dried residues were dissolved in 100 µL methanol and vortex-mixed twice, with 10 min rest at room temperature in between, and 10 µL were injected into the HPLC.

Xanthohumol was chromatographed on a JASCO HPLC system (LC Net II ADC, AS-2059-SF Plus, PU-2080 Plus, Intelligent Column Thermostat CO-2060 Plus, DG-2080-53, LG-2080-02 and a photodiode array detector PDA MD-2018; JASCO, Groβ-Umstadt, Germany) equipped with a Kinetex PFP column (Kinetex PFP, 100 Å pore size, 250 × 4.6 mm (length × diameter), 5 μm particle size; Phenomenex) maintained at 35 °C. Mobile phase A (deionized water with 5% formic acid) and mobile phase B (acetonitrile with 10% deionized water and 5% formic acid) were delivered at a flow rate of 1.0 mL/min following a multistep gradient method (0 min 0% B, 1 min 30% B, 4 min 30% B, 12 min 75% B, 14 min 100% B, 20 min 30% B). The eluent was monitored by photodiode array detection at 292 nm with spectra obtained over the 220 to 850 nm range. Peaks were recorded and integrated using ChromNav version 1.19.1 (JASCO) and quantified by comparing the peak areas to those of authentic external standards.

### 2.8. Statistical Analysis

Results are expressed as mean ± SEM. Comparisons between groups were performed using the unpaired Student’s *t*-test or one-way ANOVA, as appropriate. A *p*-value <0.05 was considered statistically significant. All calculations were carried out using the statistical computer package GraphPad Prism version 4.00 for Windows (GraphPad Software, San Diego, CA, USA).

## 3. Results

### 3.1. Effects of Solubilized Xanthohumol on Body Weight Gain in Mice Fed with a Western-Type Diet (WTD)

For this study, we orally administered a native [92% *m*/*m*, n-XN] or micellar xanthohumol formulation [Xantho-Flav-Solubilisate (EW0192/B, 10% *v*/*v*, s-XN)] to mice fed a Western-type diet (WTD). We have previously shown that mice fed with this diet develop key components of the metabolic syndrome and their liver pathology mimics critical pathological steps of human NAFLD [17,28]. Mice were divided into 4 groups, and then, 3 groups of mice were fed with WTD. One group of mice received standard chow and served as control.

**Figure 1:**
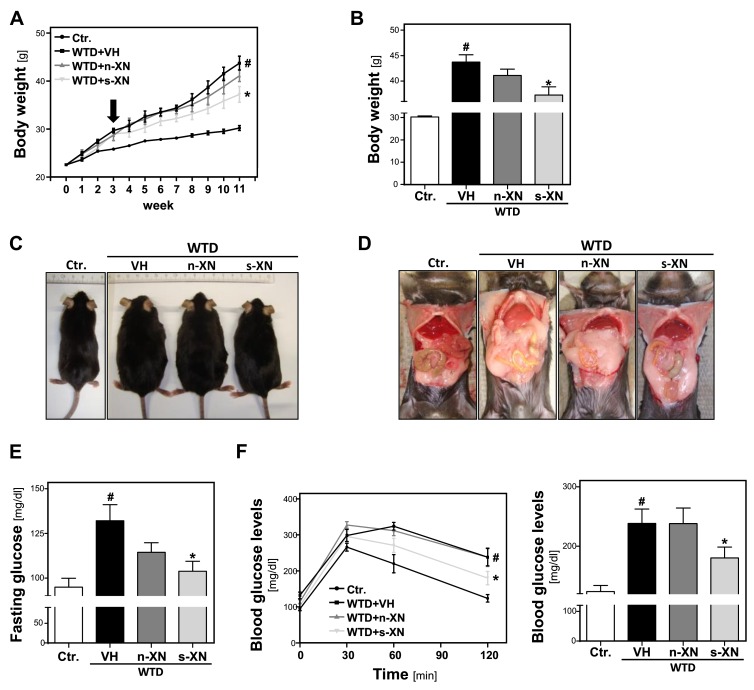
Effects of solubilized xanthohumol on body weight gain and serum glucose levels in mice fed with a Western-type diet (WTD). Mice were fed either with control diet or a Western-type diet (WTD) for three weeks. Subsequently, WTD-fed mice were treated with either n-XN or s-XN or vehicle (VH) per oral gavage daily for additional 8 weeks. (**a**) Body weight during the experiment. Arrow indicates start of treatment. (**b**) Body weight 11 weeks after start of WTD feeding (i.e., 8 weeks after the beginning of the s-XN or n-XN application). (**c**) Representative pictures of mice after 11 weeks of control or WTD-feeding. (**d**) Laparotomy showing massively increased visceral and subcutaneous white adipose tissue depots of WTD compared with the Ctr. or XN-treated groups. (**e**) Fasting Glucose levels. (**f**) Glucose levels derived from a glucose tolerance test over the time (left panel) and after 120 min (right panel). (*: *p* < 0.05 in comparison with vehicle (VH); #: *p* < 0.05 in comparison with Ctr.).

After the start of WTD-feeding, body weight (BW) gradually increased compared to control mice (Figure 1a).

After 3 weeks, BW was already significantly increased (25.8 ± 0.7 g vs. 29.2 ± 1.9; *p* = 0.0001). At this time, we started to treat 2 groups of WTD-fed animals with either s-XN or n-XN in a daily dose of 2.5 mg/kg BW by oral gavage. The third group of WTD-fed mice (WTD-VH) and control mice (CTR) received vehicle (VH) only. In the WTD-VH group, the body weight continued to increase significantly over time as compared with mice fed control chow (Figure 1a). After 11 weeks, WTD-fed mice appeared obese and showed a marked enlargement of both visceral and subcutaneous adipose tissue (Figure 1c,d). This body weight gain was significantly inhibited by application of s-XN, while n-XN exhibited only a slight effect (Figure 1a,b). Also, enlargement of adipose tissue appeared less pronounced in WTD-mice receiving s-XN (Figure 1d). No significant differences were found between treatment groups regarding food and fluid intake throughout the study (data not shown).

Feeding the WTD also led to significant higher fasting glucose and an impairment of glucose tolerance compared with mice fed with standard control chow pointing to manifest insulin resistance (Figure 1e,f). In mice that received solubilized XN (s-XN) elevated fasting glucose levels and impairment of glucose tolerance were significantly improved, while native XN (n-XN) exhibited no or only a slight effect (Figure 1e,f). In summary, s-XN application significantly ameliorated WTD-induced obesity and insulin resistance.

### 3.2. Serum Concentrations of Xanthohumol (XN) in Mice Fed with a Western-Type Diet

Xanthohumol pharmacokinetic studies in rodents and men have reported that maximum blood concentrations of xanthohumol are reached 2–4 h after oral ingestion [16,29]. Therefore, in the present study, the last oral gavage of (2.5 mg/kg BW) micellar XN (s-XN) or native XN (n-XN) was performed 3 h before termination of the experimental animals and collection of blood. Serum xanthohumol concentrations were between 104–323 nmol/L (mean, 166 nmol/L) in mice treated with s-XN, whereas no xanthohumol was detectable (limit of detection, 2.3 nmol/L) in the serum of n-XN-treated mice and the chow- or WTD-fed control animals. Although this study was not designed to determine the pharmacokinetics of the xanthohumol formulations, these data indicate that the micellar xanthohumol formulation significantly improved the oral bioavailability of xanthohumol compared to the native form in (WTD-fed) mice.

### 3.3. Effects of Solubilized Xanthohumol on Hepatic Steatosis, Inflammation and Fibrosis in Mice Fed with a Western-Type Diet

**Figure 2:**
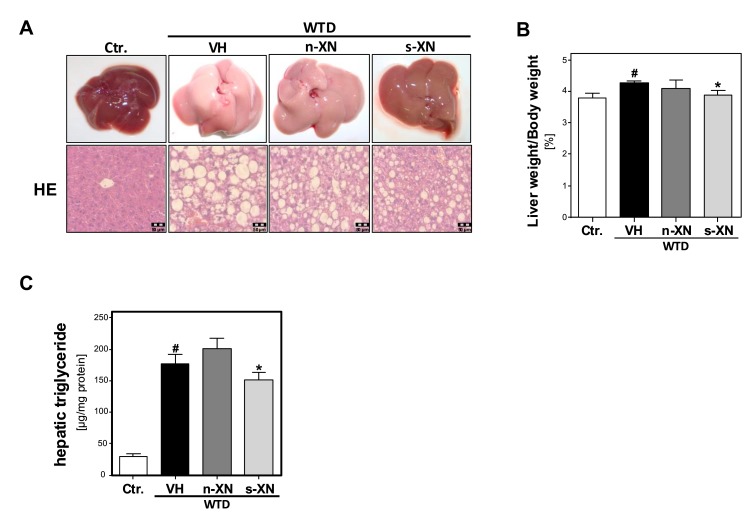
Effect of solubilized xanthohumol on hepatic steatosis in mice fed with a Western-type diet. Mice were fed either with control diet or a Western-type diet (WTD) for three weeks. Subsequently, WTD-fed mice were treated with either n-XN or s-XN or vehicle (VH) per oral gavage daily for additional 8 weeks. (**a**) Macroscopic images (upper pictures) and histological staining (hematoxylin/eosin; lower pictures) of the livers. (**b**) Liver to body weight ratio. (**c**) Hepatic triglyceride (TG) content normalized to total hepatic protein. (*: *p* < 0.05 in comparison with vehicle (VH); #: *p* < 0.05 in comparison with Ctr.).

Next, we analyzed the impact of s-XN or n-XN on WTD-induced changes in hepatic lipid accumulation. WTD-fed mice revealed remarkable hepatomegaly (Figure 2a) and a significantly increased liver weight to body weight ratio (Figure 2b) compared with control mice. Macroscopic liver appearance in the WTD group was indicative for hepatic steatosis (Figure 2a). Histological analysis showed significant mixed micro- and macrovesicular steatosis (Figure 2a). In line with this, biochemical analysis of liver tissues confirmed significantly elevated hepatic triglycerides levels in WTD-fed mice compared with controls (Figure 2c). These WTD-induced alterations were significantly reduced in mice treated with s-XN (Figure 2a–c), but not in mice given n-XN (Figure 2a–c).

**Figure 3:**
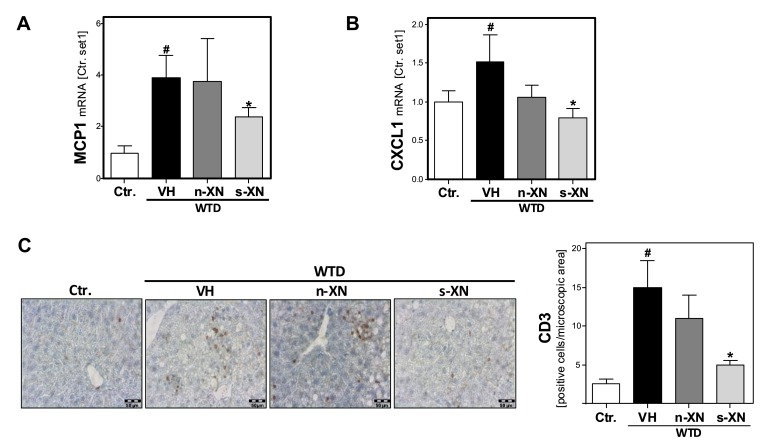
Effect of solubilized xanthohumol on hepatic inflammation in mice fed with a Western-type diet. Mice were fed either with control diet or a Western-type diet (WTD) for three weeks. Subsequently, WTD-fed mice were treated with either n-XN or s-XN or vehicle (VH) per oral gavage daily for additional 8 weeks. mRNA levels of (**a**) MCP1 (CCL2) and (**b**) CXCL1 analyzed by quantitative RT-PCR. (**c**) Microscopic images of CD3 immunohistochemical staining of liver tissue (left panel) and quantification of CD3-stained cells (right panel) (*: *p* < 0.05 in comparison with vehicle (VH); #: *p* < 0.05 in comparison with Ctr.).

Hepatic inflammation constitutes the second pathological step in NAFLD progression. WTD-feeding caused a marked increase of the hepatic expression of the chemokine monocyte chemotactic protein 1 (MCP-1) and the cytokine (CXCL1) compared to control mice (Figure 3a,b). Both pro-inflammatory factors have been shown to play a crucial role in NASH development and progression [30]. Notably, the WTD-induced expression of these pro-inflammatory factors was significantly reduced or completely abrogated, respectively, by s-XN application (Figure 3a,b). In contrast, n-XN effects on MCP-1 and CXCL1-expression did not reach the level of significance (Figure 3a,b). Moreover, CD3-immunohitochemical analysis confirmed hepatic infiltration with inflammatory cells in WTD-fed mice, which was almost completely inhibited by s-XN application (Figure 3c). In contrast, n-XN hat only slight effects (Figure 3c) on WTD-induced hepatic inflammation.

**Figure 4:**
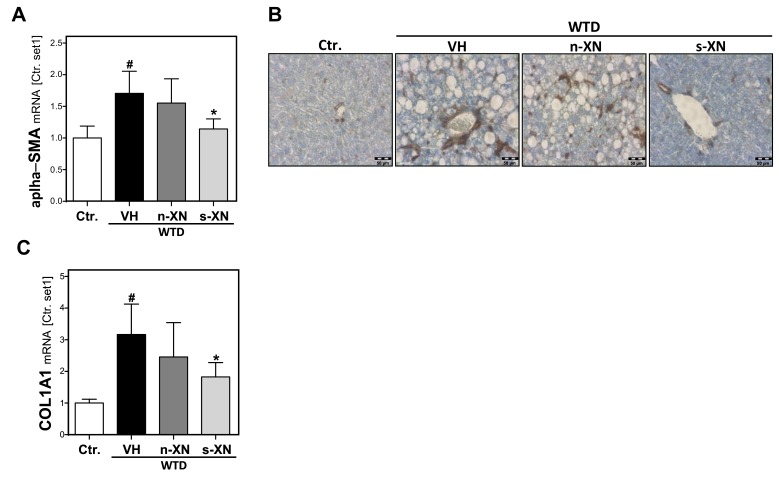
Effect of solubilized xanthohumol on hepatic fibrosis in mice fed with a Western-type diet. Mice were fed either with control diet or a Western-type diet (WTD) for three weeks. Subsequently, WTD-fed mice were treated with either n-XN or s-XN or vehicle (VH) per oral gavage daily for an additional 8 weeks. (**a**) Hepatic mRNA levels of α-SMA analyzed by quantitative RT-PCR. (**b**) Microscopic images of α-SMA immunohistochemical staining of liver tissue. (**c**) Hepatic COL1A1 mRNA analyzed by quantitative RT-PCR (*: *p* < 0.05 in comparison with vehicle (VH); #: *p* < 0.05 in comparison with Ctr.).

Sustained hepatocellular injury and inflammation can lead to hepatic fibrosis. The activation of hepatic stellate cells (HSC) is the key event of hepatic fibrosis [31]. Also in NAFLD, activated HSC are the major cellular source of extracellular matrix (ECM) deposition [32]. Histological hematoxylin and eosin (H&E) staining did not yet show prominent hepatic fibrosis in mice fed with WTD-inducing NASH diet (Figure 2a). However, quantitative qPCR analysis of the livers of WTD-fed mice revealed a markedly increased expression of alpha-smooth muscle actin (α-SMA) (Figure 4a), an established marker of activated HSC [32]. Immunohistochemical α-SMA analysis confirmed that WTD caused a marked activation of HSC (Figure 4b). This was accompanied by significantly elevated hepatic levels of collagen type I (COL1A1) in the liver of WTD-fed mice compared with control mice (Figure 4c). Collagen type I is quantitatively and qualitatively the most important extracellular matrix protein in liver fibrosis [33]. Notably, s-XN treatment significantly inhibited WTD-induced α-SMA and collagen I expression while n-XN application had only slight effects (Figure 4a–c). In summary, application of s-XN significantly reduced hepatic steatosis, inflammation and fibrosis in a WTD induced murine NAFLD-model.

## 4. Discussion

The aim of this study was to investigate the effect of micellar solubilization of xanthohumol (s-XN) in a diet-induced preclinical model of obesity, insulin resistance and NAFLD [17]. This model is based on feeding a Western-type diet (WTD), consisting of a high fat, cholesterol and fructose content. It has been increasingly recognized that besides lipid intake, also excessive cholesterol and fructose consumption significantly contribute to the development and progression of different components of the metabolic syndrome and NAFLD. Importantly, we have previously shown that feeding this WTD to mice does not only induce obesity, diabetes and dyslipidemia but also pathological changes in the liver that closely mimic liver pathology observed in patients with NAFLD [17,34].

In the present study, a WTD was fed for 3 weeks before oral administration of xanthohumol was started. At this time, the mice had already significantly elevated body-weight as compared to mice fed a control chow. Furthermore, we know from previous studies with this diet that at this time point already significantly elevated hepatic triglyceride levels and beginning hepatic inflammation had been developed (unpublished observations). Hence, the experimental setting of xanthohumol application simulated the typical timing of a potential clinical intervention in an obese individual with beginning NAFLD. Both the micellar solubilizate as well as the native extract have been applied via oral gavage to guarantee exact dosing and comparability. Still, it has to be mentioned that gavage is not physiological and further studies including different types and frequency of administration are planned in order to translate the application to future human studies.

The micellar solubilizate of xanthohumol was applied at a daily dose as low as 2.5 mg/kg BW, which is more than 10-fold lower than doses applied in previous studies showing improvement in metabolic syndrome and NAFLD symptoms. This was done based on previous studies successfully employing micellar solubilization to improve the bioavailability of other polyphenols [18,19,20,21]. Indeed, xanthohumol was detectable only in the serum of mice treated with the micellar solubilization, but not in mice receiving the native form. In one in vivo study of Legette et al., native xanthohumol was dissolved in a self-emulsifying isotropic mixture of oleic acid, propylene glycol, and Tween 80 in order to improve the bioavailability of xanthohumol. In this study, the maximum xanthohumol concentration (C_max_) was approximately 0.3 µmol/L for the highest xanthohumol dose (16.9 mg/kg BW) in rats following oral administration T_max_ (~4 h post dosing) [29]. In the present study, we detected xanthohumol serum concentrations between 0.1–0.33 µmol/L after 3 h of the last application of micellar xanthohumol solubilization (2.5 mg/kg BW) via oral gavage. Herewith, similar plasma concentrations have been achieved to those observed in the study of Legette et al. [29] after application of an approximately 12-folds higher dose (16.9 mg/kg BW). (Oral dose ratio can be calculated using allometric interspecies scaling. For example, to convert a rat dose (mg/kg BW) to the human or mouse equivalent dose, the rat dose should be either multiplied by 0.162 or 2, respectively [35]). Importantly, the elegant studies by Legette et al. [29] demonstrated also the similarity of XN metabolisms and pharmacokinetics between rodents and humans, which allows the translation of data generated in murine and rat models into clinical studies/application. Doses administered in the studies of Legette et al. (1.86, 5.64, 16.9 mg/kg BW) corresponded to scaled values of 20, 60, 180 mg XN in humans with a body weight of 66 kg [16,29]. Although our study was not designed to analyze the pharmacokinetics of the micellar XN solubilization, the data of the present study and the study from Legette et al. [29] coupled with interspecies scaling could form the basis for the dosing regimen for future clinical studies.

Fitting to the improved bioavailability, only the administration of micellar XN significantly inhibited the WTD-induced weight gain and improved glucose tolerance in mice, whereas native XN was ineffective. In agreement with this, xanthohumol at a dose of 30 mg/kg/day had no significant effect on bodyweight gain in a high-fat diet-induced mouse model [36]. Furthermore, xanthohumol application for 5 weeks in doses as high as 250–750 mg/kg/day did not exhibit significant beneficial effects on body weight gain in KK-A^y^ mice [37]. Also in obese male Zucker fa/fa rats, application of xanthohumol in a dose of 5.64 mg/kg/day for 6 weeks did not significantly affect the body weight gain [10].

The micellar xanthohumol solubilizate also significantly inhibited the WTD-induced hepatic steatosis, inflammation and fibrosis. In contrast, the native xanthohumol extract exhibited no or only marginal effects on NAFLD-pathology, which is in line with previous studies showing that significantly higher doses (of native) xanthohumol have been required to inhibit hepatic steatosis, inflammation and fibrosis in different preclinical models. Beneficial effects on hepatic steatosis in mice have been reported when xanthohumol was applied in doses of 150–750 mg/kg/day for ca. 5–8 weeks [37,38,39]. In rats fed with high-fat diet, application of 200 mg xanthohumol/kg/day for 6 weeks significantly inhibited the hepatic steatosis [40]. In contrast, doses up 16.9 mg/kg/day did not show any beneficial effect on hepatic triglyceride concentrations in Zucker fa/fa rats [10]. Furthermore, we and others have shown that xanthohumol doses as high as 100–500 mg/kg/day BW significantly inhibited hepatic inflammation and fibrosis in different models of chronic liver injury [12,13,14,15].

Together, these data show that the bioavailability and accordingly also the biological efficacy of XN was significantly improved by the micellar solubilization. This is in line with previous studies demonstrating enhanced oral bioavailability of micellar curcumin or a trans-resveratrol from a grapevine-shoot extract [18,19,20,21]. Thus, micellar solubilization may overcome the problem of poor bioavailability of some nutrients/polyphenols and facilitate their application for the prevention and treatment of metabolic diseases. Moreover, our study reinforces xanthohumol as a very promising agent for the improvement of different components of the metabolic syndrome including all pathological steps of NAFLD. Importantly and to the best of our knowledge, this is also the first study demonstrating beneficial effects of xanthohumol not only in the prevention, but also in the treatment of metabolic disease states.
